# Alarplasty and its effects on respiratory function: a cross-sectional study

**DOI:** 10.3389/fmed.2026.1776098

**Published:** 2026-04-07

**Authors:** Mohammad Z. Darabseh, Aseel Aburub, Rama Al-rawajfeh, Lubna Khreesha, Khaled M. Bani Hani, Asma Albtoosh, Viktória Prémusz, Pongrác Ács

**Affiliations:** 1 Department of Physiotherapy, School of Rehabilitation Sciences, The University of Jordan, Amman, Jordan; 2 Department of Physiotherapy, Faculty of Allied Medical Sciences, Applied Science Private University, Amman, Jordan; 3 Department of Special Surgery, School of Medicine, The University of Jordan, Amman, Jordan; 4 Queen Alia Heart Institute, Royal Medical Services, Amman, Jordan; 5 Department of Internal Medicine, Respiratory and Sleep Medicine, School of Medicine, The University of Jordan, Amman, Jordan; 6 Institute of Physiotherapy and Sports Science, Faculty of Health Sciences, University of Pécs, Pécs, Hungary

**Keywords:** alarplasty, nasal airflow, pulmonary function, respiratory muscle strength, spirometry

## Abstract

**Background:**

Alarplasty is a cosmetic procedure modifying nostril dimensions, but its long-term physiological effects on pulmonary function remain unclear. This study assessed pulmonary parameters to explore potential functional implications.

**Methods:**

A cross-sectional study included 22 women who had undergone alarplasty at least two years prior. Spirometry measured FEV1, FVC, and FEV1/FVC ratio. Respiratory muscle strength was evaluated using maximal inspiratory pressure (MIP) and maximal expiratory pressure (MEP). Results were compared to predicted normative values, and correlations with time since surgery were analyzed.

**Results:**

FEV1 predicted % and FEV1/FVC ratio were significantly lower than expected (*p* < 0.05), suggesting airflow limitations. MIP and MEP were also reduced (*p* ≤ 0.005), indicating weaker respiratory muscles. A significant negative correlation was found between years since surgery and FEV1 (*p* = 0.002), FVC (*p* = 0.0001), and FEV1 predicted % (*p* = 0.0002).

**Conclusion:**

Previous alarplasty may be associated with differences in pulmonary function parameters. Further studies with larger samples are needed to confirm these findings.

## Introduction

The increasing prevalence of elective cosmetic procedures has brought attention to the need for understanding their implications beyond aesthetic outcomes (1). Alarplasty, a surgical procedure aimed at modifying the size and shape of the nostrils, is among the commonly performed facial surgeries (2). While its primary objective is to enhance facial harmony and meet patient preferences, the potential functional consequences of altering nasal anatomy have not been extensively investigated.

Nasal breathing plays a pivotal role in respiratory physiology, contributing to effective oxygen exchange and maintaining airway resistance within optimal ranges (3). The nostrils, as the initial gateway for inhaled air, serve as regulators of airflow and resistance, significantly impacting respiratory efficiency (3). Surgical alterations of nostril dimensions, as performed in alarplasty, may inadvertently affect these physiological mechanisms (4). Despite the widespread acceptance of alarplasty as a safe and effective procedure, its potential impact on pulmonary function remains understudied. Understanding these effects is crucial not only for optimizing surgical techniques but also for ensuring patient safety and satisfaction (4).

The implications of nasal airflow dynamics extend beyond mere respiratory efficiency, as alterations in nostril structure can influence the development of long-term respiratory conditions (3, 4). For instance, studies have highlighted that reduced nasal patency may lead to compensatory mouth breathing, which is associated with increased upper airway resistance and a higher risk of obstructive sleep apnea (5, 6). Furthermore, chronic alterations in airflow patterns can potentially disrupt the delicate balance of humidification and filtration processes within the nasal cavity, predisposing individuals to respiratory tract infections and reduced mucociliary clearance (5, 6). Given these critical considerations, it is essential to investigate whether alarplasty, despite its cosmetic intent, poses any latent risks to respiratory health (7). Identifying such risks can guide the development of procedural safeguards and postoperative monitoring protocols, ensuring that the functional integrity of nasal anatomy is preserved (5, 6).

Although alarplasty is gaining popularity, especially in Middle Eastern and Asian populations due to aesthetic preferences, few studies have investigated the long-term functional implications of isolated nostril modification procedures. Most available evidence regarding functional nasal surgery has focused on rhinoplasty and septoplasty, where postoperative alterations in nasal airflow dynamics and airway resistance have been reported. Previous investigations into rhinoplasty and septoplasty suggest potential changes in nasal airflow and pulmonary efficiency, highlighting a need to investigate whether similar outcomes may also be observed following alarplasty (8–10). Previous investigations into rhinoplasty and septoplasty suggest potential changes in nasal airflow and pulmonary efficiency, highlighting a need to investigate whether similar outcomes are observed following alarplasty. The primary objective of this study was to explore possible associations between previous aesthetic alarplasty and pulmonary function parameters in adult women. By employing rigorous assessments, including spirometry, this research seeks to provide evidence-based insights into how modifications to nasal anatomy might influence respiratory capacity.

## Method

### Study design

A cross-sectional study was conducted to achieve the study goal. Ethical approval was obtained from the Research Ethics Committee at the Faculty of Allied Medical Sciences at Applied Science Private University (reference no: AMS-2025-2).

### Recruitment and sample size

Participants were recruited through online invitations to participate in this study which were posted in social media (Facebook and Instagram) to enhance recruitment. Due to the limited information about number of people who had conducted the alarplasty surgeries in Jordan, no sample size calculations were conducted. Informed consent was obtained from all subjects involved in the study. Participants provided written consent after being informed about the study aims, procedures, and potential risks.

Thirty-five individuals expressed interest in participation. After screening for eligibility criteria, 9 individuals were excluded due to additional nasal surgical procedures or medical conditions affecting respiratory function. Twenty-six eligible participants were invited for testing, of whom 22 completed all study procedures and were included in the final analysis.

### Exclusion and inclusion criteria

Participants were included if they: have conducted alarplasty at least two years before the start of the study; above 18 years old. Participants were excluded if they: have any diagnosed cardiorespiratory, musculoskeletal, or neurological conditions; or had any other surgeries that could limit their exercise capacity or ability to perform spirometry. Participants included in the study reported having undergone isolated aesthetic alar base reduction procedures (alarplasty) performed for cosmetic purposes. Individuals who reported undergoing concurrent rhinoplasty, septoplasty, or other nasal reconstructive procedures during the same surgical session were excluded from participation. Participants were screened using a brief health questionnaire to identify any history of chronic respiratory disease, including asthma, chronic obstructive pulmonary disease (COPD), or other pulmonary disorders. Individuals reporting physician-diagnosed respiratory disease or ongoing respiratory symptoms were excluded from participation.

### Outcome measures

#### Pulmonary function test

The spirometry was assessed using a spirometer (BTL Spirometry, United Kingdom) and in accordance with the European Respiratory Society/American Thoracic Society (ERS/ATS) guidelines (11). The best of three consecutive blows was used to obtain the FEV1 (forced expiratory volume in the first second), FVC (Forced vital capacity) and FEV1/FVC ratio (11). Results were compared with norms from the Global Lung Initiative, to get the predicted percent values (12). Participants were tested in a quiet, temperature-controlled laboratory environment. The spirometer (BTL Spirometry, United Kingdom) was calibrated before each session following ERS/ATS guidelines. All pulmonary tests were conducted with participants seated and using a nose clip. For MIP and MEP, a digital pressure manometer (BTL Spirometry, United Kingdom) was used according to standard European Respiratory Society/American Thoracic Society (ERS/ATS) guidelines (11) and acceptability and repeatability criteria. Participants performed a minimum of three acceptable maneuvers and additional attempts were allowed if necessary to obtain reproducible results. All measurements were performed by a trained physiotherapist experienced in pulmonary function testing. Predicted spirometry values were calculated using the Global Lung Function Initiative (GLI-2012) reference equations. The spirometer software automatically generated predicted values using the “Other/Middle Eastern” ethnicity category. Results were expressed as percentage predicted values rather than z-scores.

### Statistical analysis

Descriptive statistics were used to characterize participants based on the studied variables using SPSS software. Mean and standard deviation were used to describe continuous variables, while frequency and percentage were used for categorical data. Continuous variables included age, BMI, FEV1, FVC, the FEV1/FVC ratio, and duration since surgery. Categorical variables included sex assigned at birth, marital status, educational level, and smoking status.

Differences between FEV1, FVC, FEV1/FVC and their predicted values for people with the same age, height, weight, sex assigned at birth, and ethnic origin (according to the Global Lung Initiative data), were assessed using Paired T-test. Paired t-tests were used because predicted values generated from the GLI-2012 equations are individualized estimates calculated for each participant based on age, sex, height, and ethnicity. Therefore, each participant’s measured value was compared directly with their corresponding predicted value, allowing within-subject comparison. Prior to conducting parametric tests, the normality of continuous variables was assessed using the Shapiro–Wilk test and visual inspection of distribution plots.

Ethics Approval: was obtained from the Research Ethics Committee at the Faculty of Allied Medical Sciences at Applied Science Private University (reference no: AMS-2025-2).

## Results

A total of 22 participants accepted to participate in the study. All data were collected between January 2025 until April 2025. The following are the details of the participants.

All participants were females with a mean (standard deviation) of was 37.39 (3.92) years, and BMI was 21.18 (2.95). Table 1 represents the Anthropometric results for both groups. Regarding smoking status, 18 participants (81.8%) were non-smokers and 4 participants (18.2%) reported light smoking (<5 cigarettes/day). None reported a history of heavy smoking or long-term tobacco exposure. Exploratory comparisons between smokers and non-smokers did not demonstrate statistically significant differences in pulmonary parameters; however, the small number of smokers limits meaningful subgroup analysis. Participants also reported generally low to moderate levels of physical activity, with none engaged in competitive sports or structured respiratory training programs. None of the participants reported chronic respiratory symptoms such as persistent cough, wheezing, dyspnea on exertion, or previously diagnosed pulmonary disease at the time of recruitment.

**Table 1:** Anthropometric results (*n* = 22).

Demographics	Mean ± SD
Age	37.39 ± 3.92
BMI	21.18 ± 2.95
Years since surgery	4.00 ± 2.29

Data are presented as mean ± SD.

### Pulmonary function

FEV1 (2.52 ± 0.59), FEV1 predicted % (59.56 ± 9.24%) and FEV1/FVC (57.71 ± 7.32) were significantly lower than predicted norms (*p* < 0.05) (according to the global lung initiative data) (Table 2). FVC (4.61 ± 0.72 L) showed no significant difference (*p* > 0.05).

**Table 2:** Differences between pulmonary function and their predicted normal values according to the global lung initiative.

Respiratory function	Alarplasty participants	Normal predicted value	*p*-value
FEV1 (L)	2.52 ± 0.59	3.65 ± 0.65	0.001*
FEV1pred (%)	59.56 ± 9.24	102.68 ± 22.26	0.031*
FVC (L)	4.61 ± 0.72	4.92 ± 0.54	0.289
FVCpred (%)	89.46 ± 7.24	104.26 ± 12.15	0.155
FEV1/FVC	57.71 ± 7.32	83.84 ± 5.42	0.035*

Data are presented as mean ± SD; FEV1: Forced expiratory volume in one second; FVC: Forced vital capacity, *p value < 0.05.

### Respiratory muscles strength

MIP (91.26 ± 11.44 cmH2O) and MEP (71.36 ± 10.17 cmH2O) were significantly lower than predicted values (*p* ≤ 0.005) (Table 3).

**Table 3:** Difference in respiratory muscles strength results (mouth pressure) in between alarplasty participants and their normal predicted values (*n* = 22).

Respiratory muscle strength	Alarplasty participants	Normal predicted value	*p*-value
MIP (cmH2O)	91.26 ± 11.44	129.19 ± 24.63	0.007**
MEP (cm H2O)	71.36 ± 10.17	189.74 ± 49.24	0.009**

Data are presented as mean ± SD; MIP Maximal inspiratory pressure; MEP Maximal expiratory pressure; cmH2O centimeter of water; **p value < 0.005.

### Correlations with years since surgery

**Figure 1:**
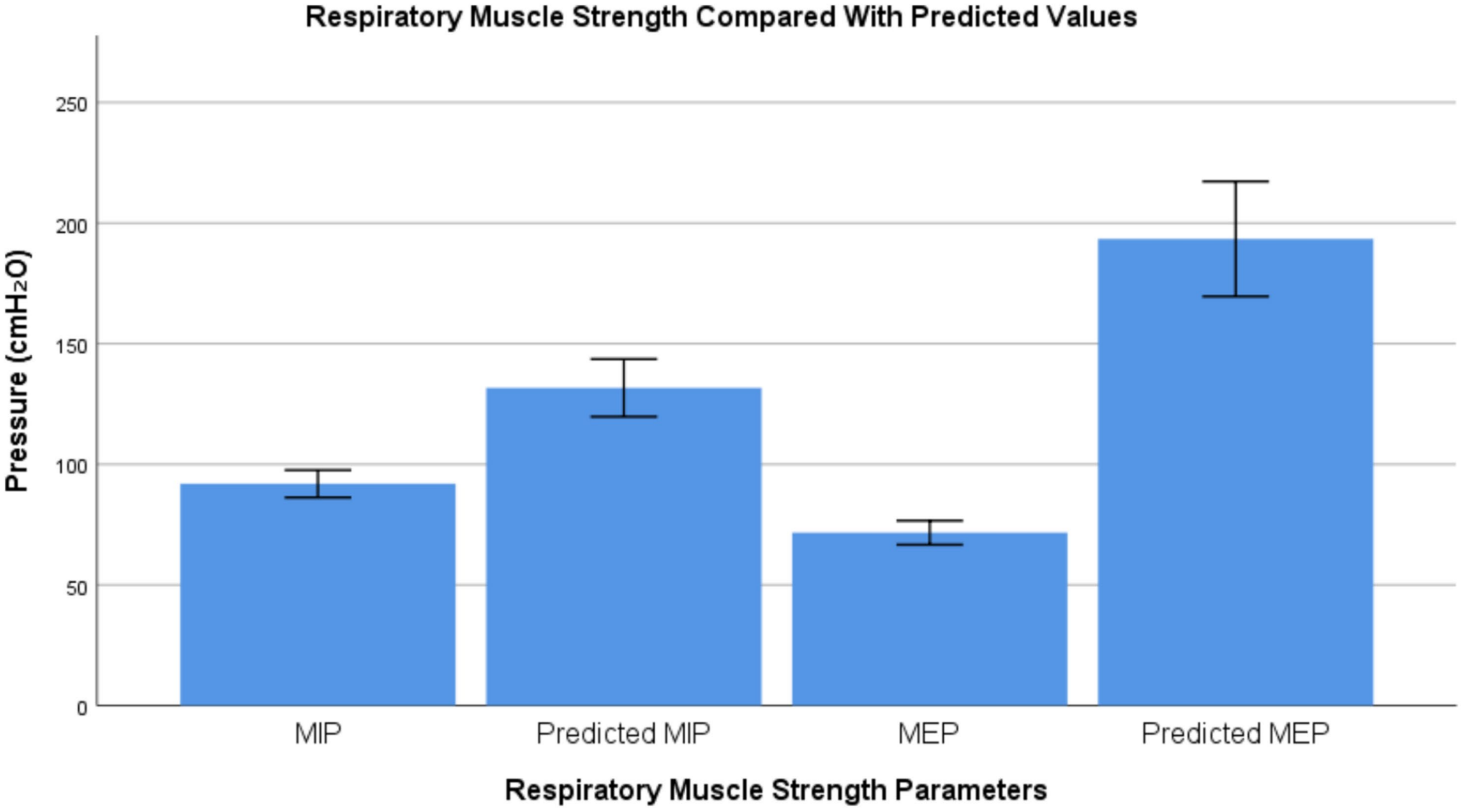
Respiratory muscle strength compared with predicted values. Bar graph illustrating maximal inspiratory pressure (MIP) and maximal expiratory pressure (MEP) measured in participants who had previously undergone alarplasty compared with their predicted normative values. Bars represent mean values and error bars indicate ±1 standard deviation (SD). Pressure values are expressed in centimeters of water (cmH_2_O).

**Figure 2:**
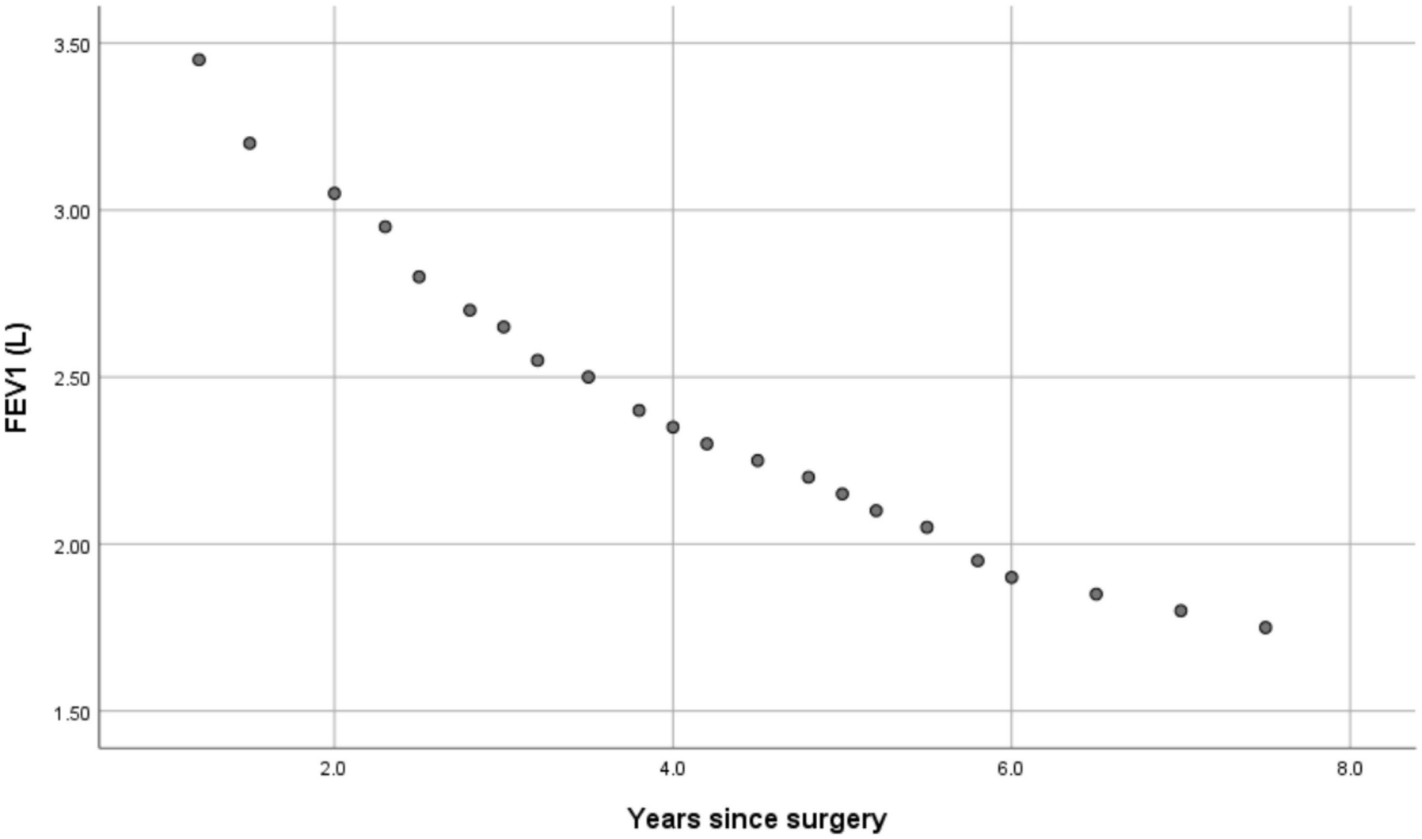
Scatter plot showing Pearson correlation between years since surgery and FEV1.

**Figure 3:**
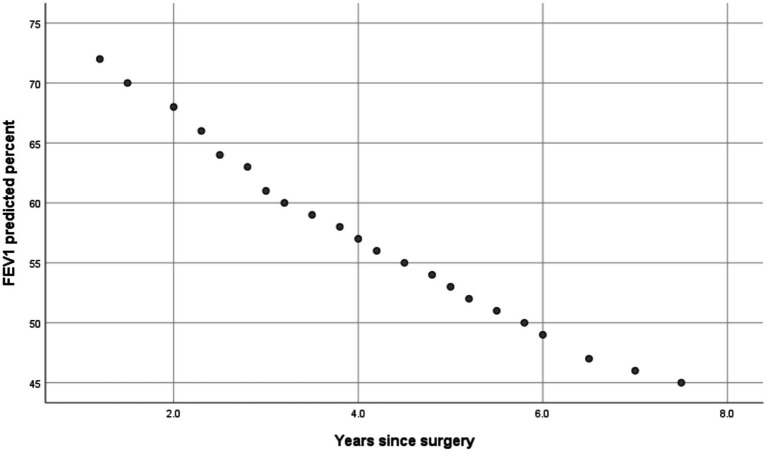
Scatter plot showing Pearson correlation between years since surgery and FEV1 predicted %.

As in Table 4, subsequent Pearson’s correlation tests showed significant negative correlations between years since surgery and: FEV1 (r = −0.921, *p* = 0.002); FVC (r = −0.392, *p* = 0.007); and FEV1 predicted % (r = −0.813, *p* = 0.002). Graphical representations of respiratory muscle strength differences and the correlations between years since surgery and spirometric parameters are presented in Figures 1–3.

**Table 4:** Correlations between years since surgery and respiratory function in alarplasty participants (*n* = 22).

Respiratory function’		Years since surgery
FEV1 (L)	R	−0.921*
	*p*-value	0.002
FEV1pred (%)	R	−0.813**
	*p*-value	0.002
FVC (L)	R	−0.392**
	*p*-value	0.007
FVCpred (%)	R	−0.443
	*p*-value	0.172
FEV1/FVC	R	−0.421
	*p*-value	0.198
PEF (L)	R	−0.394
	*p*-value	0.231
PEFpred (%)	R	−0.646*
	*p*-value	0.023
MIP (cmH2O)	R	−0.067
	*p*-value	0.844
MEP (cmH2O)	R	−0.238
	*p*-value	0.28

*Correlation is significant at the 0.05 level. **Correlation is significant at the 0.005 level.

## Discussion

To the best of our knowledge, this is the first study exploring potential associations between previous aesthetic alarplasty and pulmonary function parameters. The significant differences observed in pulmonary function and respiratory muscle strength suggest that nostril modifications may have unintended physiological consequences.

The most notable result was the lower FEV1 predicted % and FEV1/FVC ratio in individuals who had undergone alarplasty compared to their predicted normal values. These findings indicate a potential reduction in expiratory airflow, which may be attributed to subtle structural changes in nasal airflow resistance following surgery. This aligns with prior research indicating that nasal patency influences overall respiratory function, particularly in maintaining effective airflow regulation (3, 4). Alterations in nasal architecture may contribute to increased airway resistance, which, over time, could impact pulmonary function.

Interestingly, while FVC values did not show a significant difference from predicted norms, the reduction in FEV1/FVC suggests that participants may be experiencing some degree of airflow limitation. This observation warrants further investigation into whether nasal airway modifications can induce compensatory breathing patterns, such as increased mouth breathing, which has been linked to conditions like obstructive sleep apnea and upper airway resistance syndrome (5, 6). The significant correlation between years since surgery and reduced pulmonary function parameters further underscores the potential long-term impact of nostril modification on respiratory health. Although formal screening for sleep-disordered breathing was not performed, participants did not report diagnosed obstructive sleep apnea or chronic mouth breathing. However, systematic assessment of sleep-related breathing symptoms was beyond the scope of this study and represents an important direction for future research.

The observed reductions in maximal inspiratory pressure (MIP) and maximal expiratory pressure (MEP) further reinforce the notion that altered nasal resistance may influence respiratory mechanics. Reduced nasal airflow efficiency could lead to increased respiratory effort, potentially affecting respiratory muscle strength over time. This finding is particularly relevant given the role of nasal breathing in optimizing lung mechanics and oxygen exchange (7).

Several hypothetical mechanisms may explain these findings. However, because nasal airflow resistance and nasal valve function were not directly measured in this study (e.g., using rhinomanometry or other objective assessments), these explanations should be interpreted as theoretical possibilities that require confirmation in future research. It should be noted that alarplasty alters the external nasal valve, which plays a critical role in regulating nasal airflow. Even minor modifications to nasal structure may lead to increased resistance or turbulence, influencing respiratory efficiency (11). Second, chronic changes in airflow patterns may contribute to long-term functional adaptations, potentially affecting lung volumes and airway patency. Although the spirometric values suggested reduced airflow compared with predicted reference values, none of the participants reported previously diagnosed respiratory disease. These findings should therefore be interpreted cautiously and may reflect functional variations rather than clinically diagnosed obstructive pulmonary pathology.

Despite these findings, several limitations should be acknowledged. First, the cross-sectional design limits the ability to infer causal relationships between alarplasty and pulmonary function parameters. Because participants were assessed only at a single time point after surgery, it is not possible to determine whether the observed differences existed prior to the surgical procedure. Second, the study did not include a control group of individuals without alarplasty. Although comparisons with predicted values derived from the Global Lung Function Initiative provide a useful reference, these predicted values do not represent a matched control group and therefore cannot fully account for individual variability. Third, preoperative pulmonary function measurements were not available, which prevents direct evaluation of changes occurring after surgery. Finally, the relatively small sample size (*n* = 22) may limit the generalizability of the findings and increases the potential for statistical variability. Additionally, multiple statistical comparisons were performed across several pulmonary and respiratory muscle strength variables. This increases the possibility of Type I error. Future studies with larger samples and confirmatory statistical approaches are therefore warranted. Future research using longitudinal designs with pre- and postoperative measurements and appropriate control groups will be necessary to better understand the potential functional implications of alarplasty. Future research should include longitudinal follow-up to track changes over time and investigate causality. Moreover, sleep studies could determine whether airflow changes translate into sleep-related breathing disorders.

## Conclusion

In conclusion, the findings of this exploratory cross-sectional study suggest that previous alarplasty may be associated with differences in pulmonary function parameters and respiratory muscle strength. These results emphasize the need for careful preoperative assessment and postoperative monitoring to ensure that nasal modifications do not compromise respiratory efficiency. These findings may suggest that structural modifications of the nasal valve could potentially influence airflow dynamics; however, direct measurement of nasal resistance was not performed in this study. Given the relatively small sample size and cross-sectional exploratory design, these findings should be interpreted as preliminary rather than definitive evidence of physiological changes following alarplasty. Further research is warranted to develop surgical techniques that optimize cosmetic outcomes while preserving nasal function.

## Data Availability

The original contributions presented in the study are included in the article/supplementary material, further inquiries can be directed to the corresponding authors.
